# Determining Biophysical Protein Stability in Lysates by a Fast Proteolysis Assay, FASTpp

**DOI:** 10.1371/journal.pone.0046147

**Published:** 2012-10-03

**Authors:** David P. Minde, Madelon M. Maurice, Stefan G. D. Rüdiger

**Affiliations:** 1 Cellular Protein Chemistry, Bijvoet Center for Biomolecular Research, Utrecht University, Utrecht, The Netherlands; 2 Department of Cell Biology, University Medical Center Utrecht (UMCU), Utrecht, The Netherlands; University of South Florida College of Medicine, United States of America

## Abstract

The biophysical stability is an important parameter for protein activity both *in vivo* and *in vitro*. Here we propose a method to analyse thermal melting of protein domains in lysates: Fast parallel proteolysis (FASTpp). Combining unfolding by a temperature gradient in a thermal cycler with simultaneous proteolytic cleavage of the unfolded state, we probed stability of single domains in lysates. We validated FASTpp on proteins from 10 kDa to 240 kDa and monitored stabilisation and coupled folding and binding upon interaction with small-molecule ligands. Within a total reaction time of approximately 1 min, we probed subtle stability differences of point mutations with high sensitivity and in agreement with data obtained by intrinsic protein fluorescence. We anticipate a wide range of applications of FASTpp in biomedicine and protein engineering as it requires only standard laboratory equipment.

## Introduction

Protein function and activity depends on their structure and stability. Protein structure and stability are affected by various factors, such as the specific cellular environment or binding to particular ligands. For instance, some proteins need the presence of specific metals or small-molecule or protein ligands to get sufficiently stabilised to perform their biological function. Binding proteins may induce structure in proteins that lack structure in isolation such as intrinsically disordered proteins (IDPs).

Various powerful assays probe structure and stability of proteins. *In vitro* methods using purified protein include spectroscopic methods such as Circular Dichroism for secondary structure analysis, intrinsic fluorescence for tertiary structure analysis and NMR for residue-specific information. Thermal methods such as Differential Scanning Calorimetry (DSC) and Isothermal Titration Calorimetry (ITC) quantitatively determine protein stability and interactions by monitoring changes of enthalpy and entropy. Several strategies probe biophysical parameters *in vivo* or *ex vivo*, such as *in vivo* folding sensors using fluorescent proteins or fluorescent small-molecule tags or *ex vivo* pulse proteolysis [1]–[3].

Inspired by the versatility of proteolysis as a label-free method, we aimed at developing a fast and broadly applicable proteolytic assay that probes thermal protein melting *ex vivo* using common laboratory equipment. We used the thermostable protease Thermolysin (TL) which preferentially cleaves near the hydrophobic residues Phe, Leu, Ile, Val [4], [5]. TL showed sufficient specificity for unfolded states to probe protein stability in lysates within seconds. We applied the Fast parallel proteolysis (FASTpp) assay to monitor thermal unfolding of proteins ranging from 10 to 240 kDa and varying in secondary to quarternary structure. FASTpp detected stability alterations due to ligand binding and point mutations. Moreover, FASTpp can probe biophysical protein stability in cell lysates for biomedical screenings without genetic manipulation.

## Results

### FASTpp to assay protein stability

**Figure 1:**
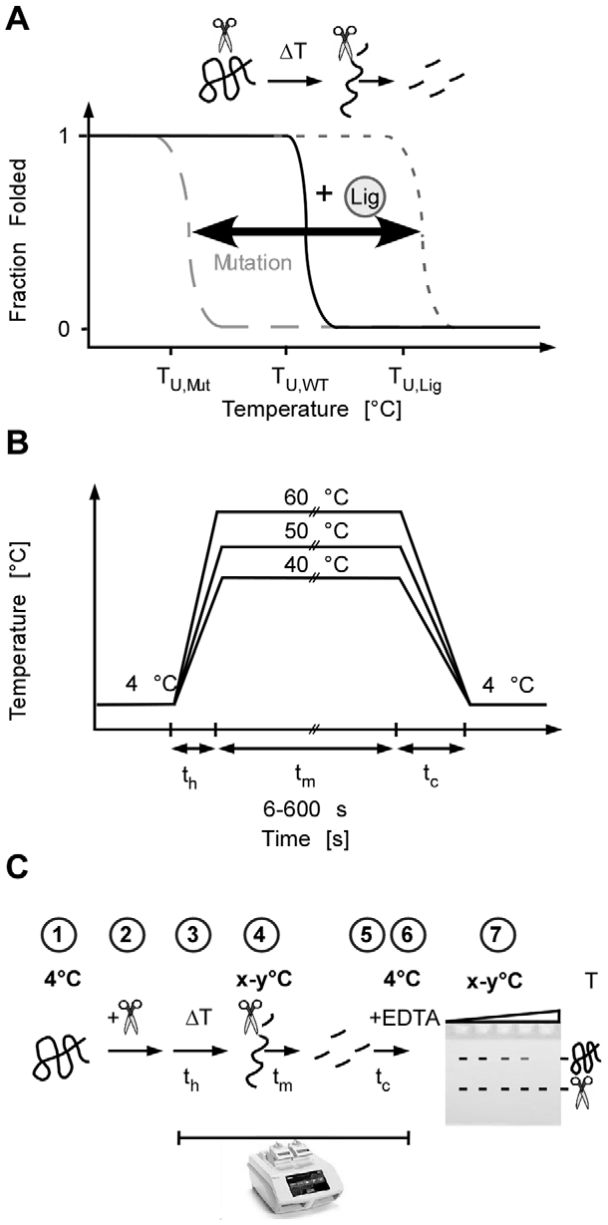
FASTpp combines automated temperature control and quantitatively characterised proteolysis to unveal protein interactions and stability. **A,** Protein stability can be probed by measuring the thermal unfolding transition in the presence of a protease. The folded state resists protease digestion while the unfolded state is readily digested on the same timescale. The thermal unfolding transition of a protein may be shifted to higher temperatures by addition of a ligand of the folded state. A shift to lower transition temperatures may occur upon destabilisation of the protein by, for instance, cancer mutations. **B,** Temperatures are controlled automatically using a standard gradient PCR setup. A mastermix of sample and protease is prepared on ice or in a cold room at 4°C and subsequently aliquoted to a PCR strip that is simultaneously heated up during the heating time **t_h_** to a range of melting temperatures that are kept for a variable melting time **t_m_**. Subsequently simultaneous cooling (cooling time, **t_c_** ) brings all aliquots back to 4°C and the reaction is quenched by addition of EDTA. **C,** Scheme of all seven processing steps of the FASTpp assay. The representation of the termocycler indicates the automated steps of the FASTpp protocol, the gel indicates the final analysis by SDS-PAGE (T, temperature; ΔT, change of temperature; x–y°C, melting temperature gradient).

The unfolding temperature of a protein serves as an intuitive indicator for protein stability. Events that affect stability also affect the unfolding temperature [6], [7]. Mutations that compromise protein structure shift, for instance, the point of thermal unfolding to lower temperatures while ligands that recognise the folded but not the unfolded state shift the thermal unfolding temperature to higher values [8]–[10] (Fig. 1A). A thermostable protease that readily cuts the unfolded but not the folded part of a protein could be used to determine the folded fraction over a wide temperature range.

Based on these considerations, we propose a fast parallel proteolysis (FASTpp) assay to determine biophysical protein stability. The principle of the method is the parallel exposure of samples of the protein of choice to a range of different temperatures, in the presence of the thermostable protease. If we choose temperatures just above and below the specific melting temperature of the protein, the temperature-dependent changes of the degradation pattern are readout for the stability of the protein. The precision of the method depends on the precise control of the heating time **t_h_**, the period for which the protein is exposed to the maximum time (melting time; **t_m_**) and the subsequent cooling down period **t_c_** (Fig. 1B).

Our assay consists of the following steps (Fig. 1C): 1. Sample preparation of the protein of interest at 4°C. 2. Addition of protease. 3. Heating time (**t_h_**) during which several aliquots of the same sample are heated up in parallel. Each aliquot reaches a specific maximal temperature; for instance the lowest sample 35°C and the highest 42°C. 4. Melting time (**t_m_**) during which aliquots are kept at defined maximum temperatures of the gradient for defined times. 5. Cooling time (**t_c_**) of the protein samples down to 4°C. 6. Stopping proteolysis by EDTA. 7. Analysis of the reaction products by SDS-PAGE. The steps 3–6 run in a thermal cycler with gradient control to ensure precision and reproducibility. Variations of **t_h_** and **t_c_** may influence the (absolute) values determined by this assay. These variables are instrument dependent, but automation ensures that all samples are reproducibly treated under identical conditions. We employed a Bio-Rad C1000 thermal cycler for which **t_h_** is e. g. 20 s for heating a sample of 10 μL from 4°C to 60°C and **t_c_** is e. g. 40 s for cooling a sample of 10 μl from 60°C to 4°C. The C1000 cycler generates a gradient spanning a temperature difference of up to 24°C in one block, which allows parallel screening of a sufficiently large temperature range for a broad range of proteins.

### Thermolysin is suitable for FASTpp

To validate this approach, we needed to identify a suitable protease, determine its cleavage rate over a broad temperature range, establish its specificity for the unfolded state and test it on a range of protein folds. We considered TL suitable due to several key features: (i) TL is thermostable up to 80°C [11]. (ii) TL preferentially cuts near exposed hydrophobic, bulky and aromatic amino acids, specifically Phe, Leu, Ala, Val and Ile [4], [5]. The preference of TL for large hydrophobic and aromatic residues ensures specificity of FASTpp. Folded proteins bury most of these amino acids inside in their hydrophobic core. Only upon unfolding, these residues are exposed and digested by TL. (iii) TL is stable over a wide pH range from 5.5 to 9 [12], it remains active in the presence of high concentrations of chaotropic reagents such as 8 M urea [1] and in the presence of EDTA-free protease inhibitors cocktails. (iv) TL is instantly inhibited by addition of EDTA, which removes TL' s essential Ca^2+^ ion [13].

**Figure 2:**
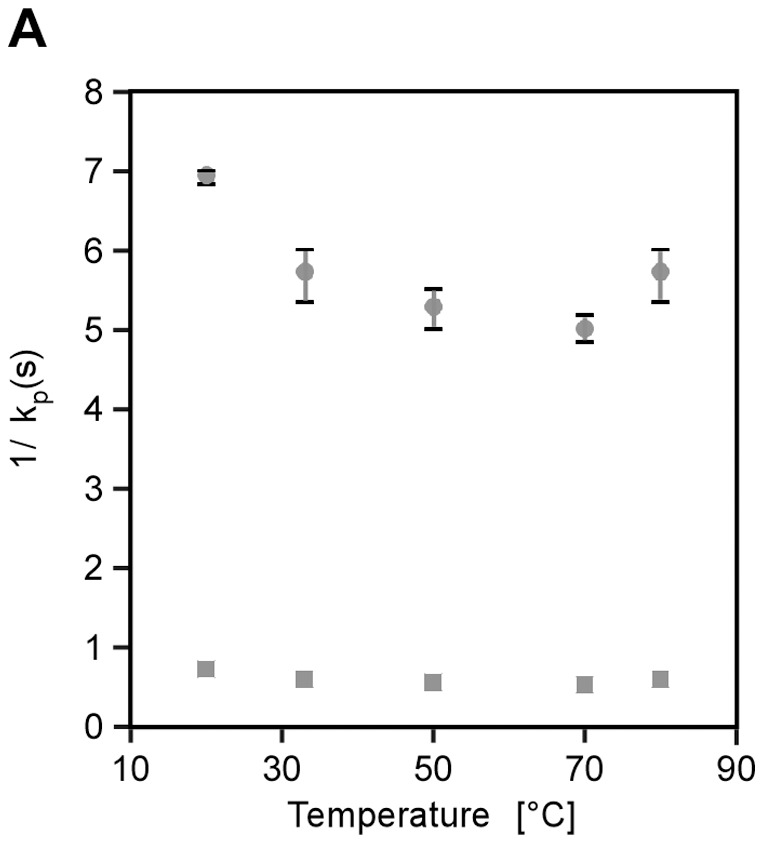
Thermolysin is active from 20°C to 80°C. Thermal dependence of intrinsic rates of proteolysis of TL from 20°C to 80°C. After extensive thermal pre-equilibration, we determined kinetic traces of cleavage of an unstructured model substrate of TL, in triplicate for each temperature. The obtained rates were extrapolated based on empirically derived formulas to final concentrations of 0.1 g/L and on top 0.01 g/L of TL [1].

As a first step we needed to validate the activity of TL under the conditions of the FASTpp experiment. We tested the temperature dependence of the proteolysis rate of TL using the unfolded peptide ABZ-Ala-Gly-Leu-Ala-NBA as established fluorogenic model substrate [1]. The fluorescence of this peptide increases upon cleavage by TL. We monitored the reaction from 20 to 80°C and for 3 to 6 nM and obtained the intrinsic rates by fitting the resulting curves to pseudo first-order kinetics as outlined in the methods section (Fig. 2) [1]. The linearly extrapolated rates varied from 1.4 to 2 s−1 at a TL concentration of 0.1 g/L, for instance 0.01 g/L TL digest 1.5 μM ABZ-Ala-Gly-Leu-Ala-NBA between 33°C and 80°C within 6 s. Remarkably, TL displayed nearly constant thermal activity over this range, rendering it suitable for FASTpp without adjusting the protease concentration for each temperature. TL's broad permissible temperature range suffices to analyse unfolding of most folded domains.

### FASTpp reveals presence of the folded state

**Figure 3:**
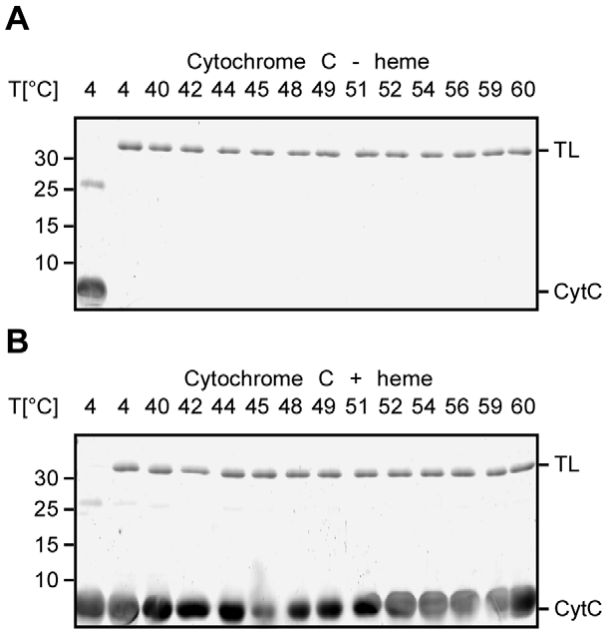
TL preferentially cleaves unfolded protein. **A,** Thermal TL resistance of *apo* Cytochrome C (CytC). Only in absence of protease at 4°C, *apo* CytC was detected on the gel. Upon addition of TL, cleavage already occured on ice and no protease resistant protein was observed after additional incubation at higher temperatures. **B,** Thermal protease resistance of CytC in presence of the bound heme ligand. A strong band of CytC is visible in absence and presence of TL from temperatures from 4°C to 60°C.

We further tested to which extent TL specifically cleaves unfolded protein chains. We investigated cytochrome C as a model substrate for TL's activity and specificity for unfolded proteins. Cytochrome C can be specifically obtained in two soluble states: either unfolded without heme or folded in the presence of heme [14], [15]. We tested whether we could distinguish both forms of cytochrome C by FASTpp. TL cleaved unfolded *apo* cytochrome C already at 4°C whereas folded, heme-bound cytochrome C was TL-resistant up to 60°C, in agreement with earlier studies [14] (Fig. 3A, B). TL digested specifically the unfolded but not the folded protein. We concluded that TL is a suitable protease for FASTpp.

### FASTpp is insensitive to variation of TL concentration

**Figure 4:**
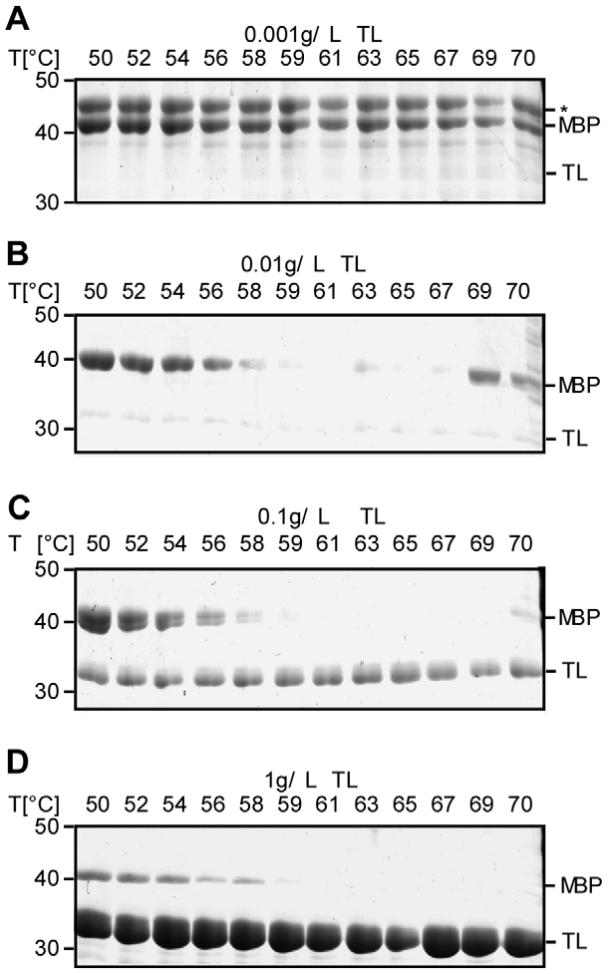
FASTpp is robust over 3 orders of magnitude of TL concentration changes. **A,** Thermal TL resistance of MBP using limiting TL concentration of 0.001 g/L. Over the entire temperature range from 50°C to 70°C, MBP remains intact. **B,** Thermal protease resistance of MBP using a TL concentration of 0.01 g/L. At this TL concentration, a clear thermal unfolding transition becomes apparent between 50°C and 60°C. Likely due to kinetic competition between irreversible aggregation and proteolytic cleavage of the unfolded state, some MBP is not digested at 69 and 70°C. **C,** Thermal protease resistance of MBP using limiting TL concentration of 0.1 g/L. A similar unfolding transition of MBP as in B is observed. **D,** Thermal protease resistance of MBP using limiting TL concentration of 1 g/L. A similar thermal unfolding transition of MBP is observed as in B.

To refine the experimental parameters, we selected Maltose Binding Protein (MBP) as a substrate for FASTpp because it is structurally well-characterised and folds both in the presence and absence of ligand. We first probed the influence of TL concentration over four orders of magnitude on the apparent thermal melting temperature of MBP using a gradient of 50°C to 70°C and constant **t_m_** (6 s) [16]. At the lowest TL concentration of 0.001 g/L, no detectable cleavage of MBP occurred (Fig. 4A). From 0.01 to 1 g/L TL (340 nM –34 μM), we observed loss of thermal proteolysis resistance at 59°C (Fig. 4B–D). Assuming comparable cleavage kinetics of the model peptide substrate and unfolded protein, we expected a minimal required cleavage time of approximately 6 s at 0.01 g/L TL to quench the unfolded fraction of protein under these conditions. Our TL titration results validated this theoretical prediction. Interestingly, at 0.01 g/L TL, we detected unfolding and concomitant cleavage of MBP at a temperature of 61°C. An uncut MBP band however remained at temperatures from 63°C to 70°C. We suspect kinetic competition between aggregation and cleavage at higher temperatures, which may protect MBP from complete cleavage because hydrophobic residues typically self-interact within aggregates. We chose a TL standard concentration of 0.1 g/L (3.4 μM) for further experiments.

### Kinetic protein stability can be probed by FASTpp at variable t_m_

**Figure 5:**
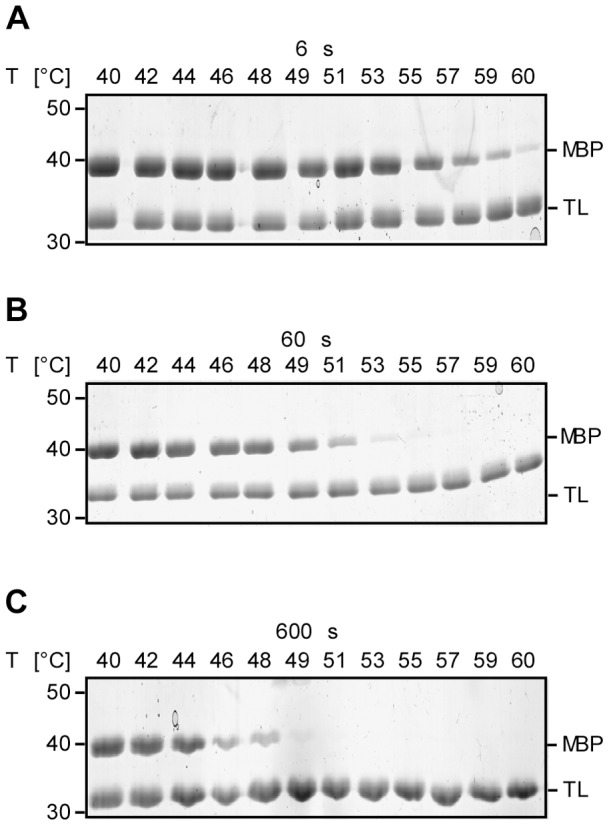
FASTpp can monitor kinetic stability of proteins by change of t_m_. **A,** Thermal TL resistance of MBP using 6 s **t_m_**. MBP was increasingly cleaved from 40°C to 60°C. **B**, Thermal TL resistance of MBP using 60 s **t_m_**. MBP was increasingly accessible to digestion from 40°C to 53°C. Above 53°C, no MBP was detected. **C,** Thermal TL resistance of MBP using 600 s t_m_. MBP was increasingly accessible to digestion from 40°C to 49°C. Above 49°C temperature, no MBP was detected.

We now investigated how the apparent thermal unfolding transition in FASTpp is affected by **t_m_**. For this we varied **t_m_** from 6 s to 600 s. In parallel with a step-wise increase in **t_m_**, MBP digestion started at successively lower temperature. For instance at **t_m_** = 6 s, the unfolding occurred at 60°C while increasing **t_m_** to 600 s lowered the unfolding temperature to 49°C (Fig. 5A–C). Because all assay parameters are kept constant except for **t_m_**, we can monitor kinetic stability with this assay. Proteins are “kinetically-stable” under conditions where the unfolding is slow relative to the measurement time. For instance, MBP is kinetically-stable at 40°C and kinetically-unstable at 60°C for all **t_m_** values we analysed.

### Ligand stabilisation can be revealed by FASTpp

**Figure 6:**
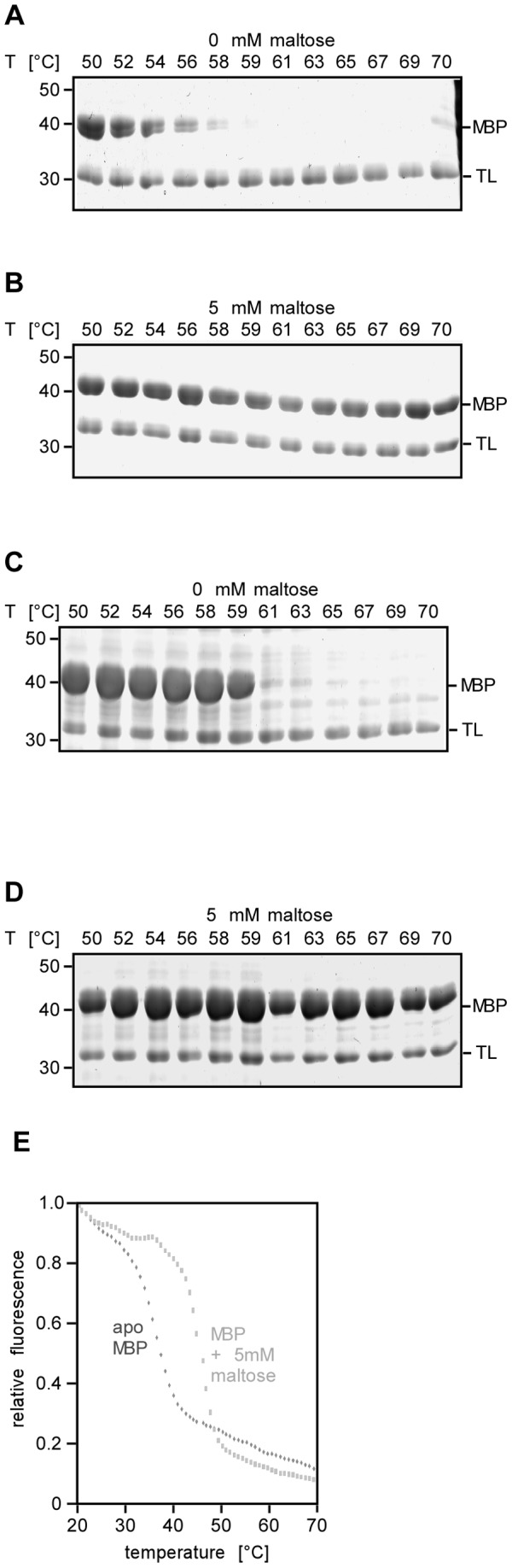
FASTpp can detect ligand effect on purified protein and in complex mixtures. **A,** FASTpp of purified MBP. Unfolding was observed above 58°C. **B,** FASTpp of purified MBP plus 5 mM maltose. Unfolding was not observed up to 70°C. **C,** FASTpp proteolysis of MBP overexpression lysate. Unfolding was observed above 59°C. **D,** FASTpp of MBP overexpression lysate plus 5 mM maltose. Unfolding was not observed up to 70°C. **E,** Fluorescence melting curves of MBP. MBP melted in absence of a ligand between 30°C and 40°C. In presence of 5 mM maltose, unfolding was observed between 40°C and 50°C.

To test the suitability of FASTpp to detect effects of ligand binding on biophysical protein stability, we analysed the influence of MBP's ligand maltose. Using a temperature range from 50 to 70°C at constant **t_m_** = 6 s, *apo* MPB became susceptible to proteolysis at 58°C whereas maltose bound MBP resisted degradation up to 70°C (Fig. 6 A, B).

We compared these FASTpp data to determining MBP's thermostability by intrinsic protein fluorescence. We observed onset of unfolding at 40°C for MBP-maltose and at 30°C for *apo* MBP, significantly lower absolute values compared to the FASTpp results (Fig. 6 A, B, E). This is possibly a result of the lower rate of temperature increase in the fluorescence experiment compared to the FASTpp experiment. The total heating time was several hours for fluorescence as compared with less than a minute in FASTpp. An alternative other explanation for discrepancies of the absolute values of thermal unfolding temperatures in both experiments could be the different contribution of secondary and tertiary structure: Fluorescence is sensitive to changes in the vicinity of tryptophanes, (i.e. typically in the core of folded proteins) and proteolysis can occur both upon loss of surface-exposed secondary structure elements or the complete tertiary structure. The stabilising effect of the maltose ligand on MBP, however, was approximately 10°C in both experiments. We therefore conclude, that FASTpp agrees qualitatively with fluorescence temperature dependence analysis about the stabilising effect of maltose on MBP (Fig. 6E). The FASTpp data confirmed a significantly stabilising effect of maltose on MBP.

### FASTpp determines protein stability in lysate

To test whether FASTpp is also suitable to assay protein stability in lysates, we compared the *in vitro* stability of MBP to the *ex vivo* stability of *E. coli* lysate overexpressing MBP. MBP resists proteolysis in lysate up to 59°C (Fig. 6C). From 61°C to 70°C, the *apo* MBP band intensity was nearly lost. In contrast, MBP's proteolytic resistance persisted up to 70°C in presence of 5 mM maltose ligand (Fig. 6D). Both for purified protein and lysate samples, maltose addition increases the unfolding temperature by more than 10°C. Interestingly, when compared with purified MBP, the *apo* MBP lysate displays a sudden unfolding transition between 59°C and 61°C while purified MBP has a much broader unfolding range between 50°C and 58°C. Lysate stabilised MBP without addition of maltose ligand. Since lysates are complex mixtures we assume that the balance of all (presumably mostly weak and transient) interactions determines the differences between biophysical stability of protein in lysates compared to experiments with purified proteins in more diluted solutions of isolated proteins [14], [17]–[21]. We conclude that FASTpp is suitable to monitor stability changes in whole cell lysates.

### BSA thermostability is not affected by maltose

**Figure 7:**
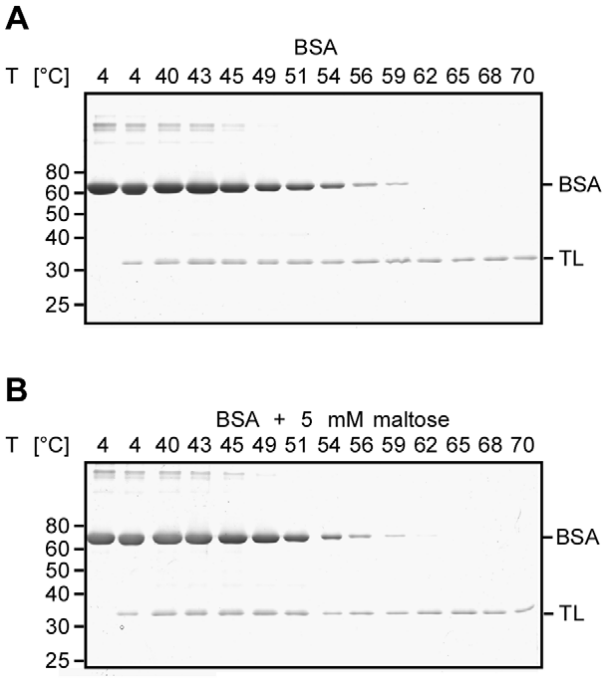
Maltose does not stabilise a control substrate that has no known maltose-binding activity. **A,** FASTpp of BSA in absence of maltose. BSA was completely digested at temperatures from 62°C to 70°C after a gradual unfolding transition over a range of temperatures from 51 to 59°C. **B,** FASTpp of BSA in presence of 5 mM maltose. BSA was completely digested at temperatures from 62°C to 70°C after a gradual unfolding transition over a range of temperatures from 51 to 59°C.

To exclude unspecific protein stabilisation by maltose, we monitored the stability of the non-maltose-binding protein BSA in the presence and absence of maltose. Maltose did not change the thermal unfolding transition of BSA in a buffer with reducing redox potential between 4°C and 59°C (Fig. 7A, B). This corroborates our conclusion that FASTpp detects specific ligand stabilisation effects.

### Large proteins assemblies can be analysed with FASTpp

**Figure 8:**
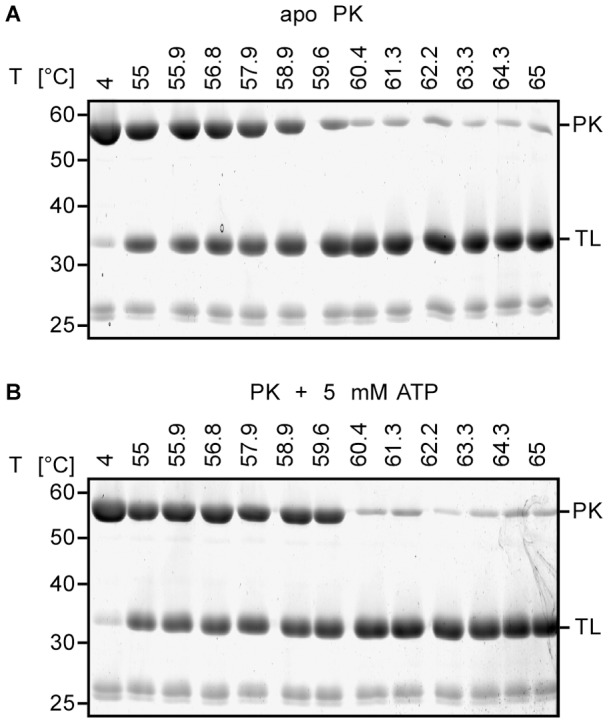
Ligand-dependent stability of a 240 kDa protein can be probed by FASTpp. **A,** Pyruvat kinase (PK) FASTpp. PK was resistant from 4°C to 58°C. A gradual decrease in the band intensity at higher temperatures indicates unfolding. Over a broad range of even higher temperatures, a small fraction of protease-resistant species persists (that likely represent aggregates formed rapidly upon unfolding). **B,** FASTpp of PK in presence of 5 mM ATP. PK was resistant against TL digestion from 4°C to 59.6°C. Already at 60.4°C, nearly complete digestion was observed.

To investigate if FASTpp is also applicable to larger proteins, we tested the 240 kDa, tetrameric Pyruvate Kinase (PK). We used a temperature range from 55 to 65°C. The protein becomes susceptible to proteolysis at 59°C (Fig. 8A, B). Surprisingly, approximately 10% of the initial PK band intensity remains at higher temperatures up to 65°C. We suspect, therefore, that thermal aggregation competes with protein cleavage above this temperature. Also, another cleavage-resistant 34 kDa fragment appears above 60°C, which might be either a more stable domain or a rapidly aggregating domain, which was protected from cleavage. We conclude that FASTpp is applicable to large multiprotein assemblies.

### FASTpp detects stability differences of point mutants

As a test case for stability discrimination of point mutants, we compared three evolved Sortase A variants that have been selected for enhanced transpeptidase kinetics: Sortase A triplemutant (3×M), Sortase A tetramutant (4×M), Sortase A pentamutant (5×M) [22].

**Figure 9:**
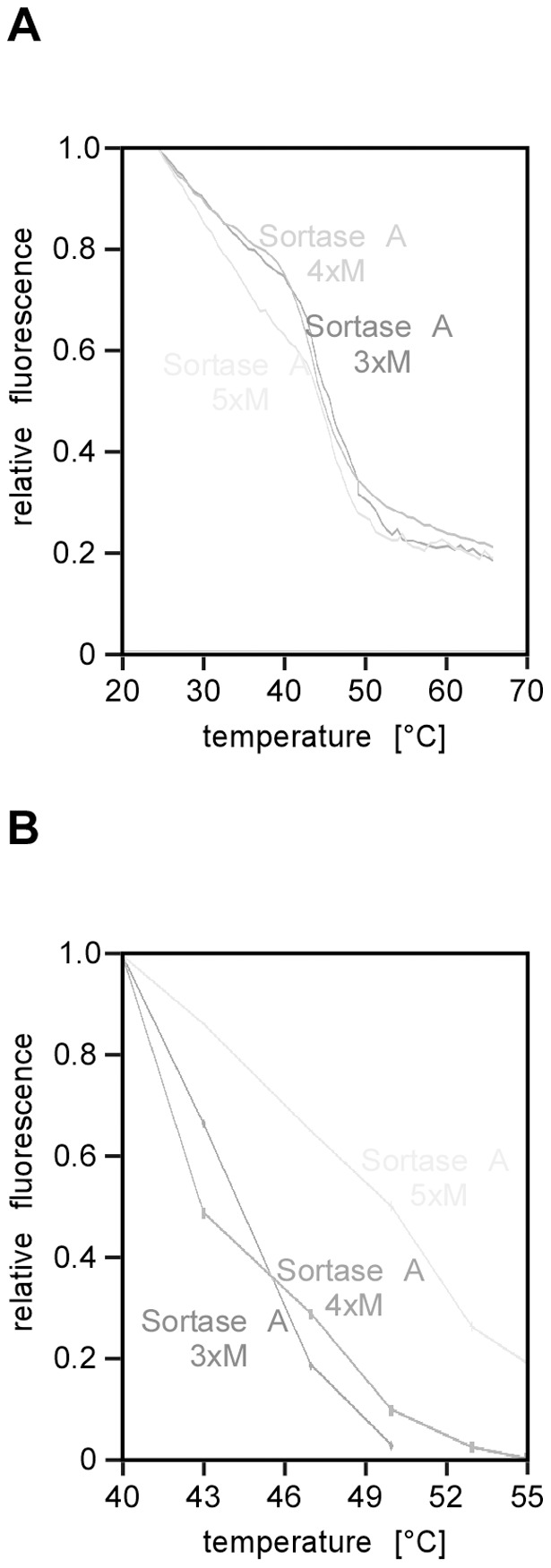
Missense mutation effects on protein stability can be probed by FASTpp. **A,** Intrinsic fluorescence temperature depence of three Sortase A variants. 3×M is triplemutant, 4×M is tetramutant, 5×M is pentamutant. **B,** FASTpp of the same three Sortase A variants as in A.

First, we analysed these variants by FASTpp. To achieve an accurate relative quantification, we made use of the strong infrared fluorescence enhancement of Coommassie dyes upon protein binding [23]. Upon quantification, we obtained the following order of stability: 3×M and 4×M are equally stable with a transition starting above 40°C; the 5×M variant displayed a less cooperative thermal unfolding transition consistent with an entropically broadened transition (Fig. 9B). Significantly more residual protein remained above 50°C for this protein variant.

Second, we used intrinsic fluorescence to probe stability differences. The variants 3×M and 4×M behaved very similar in this assay with non-linear fluorescence decay above a T_u_ of 40°C, while 5×M appeared to be slightly more stable with linear decrease continuing up to a T_u_ of 43°C (Fig. 9A). We can only achieve a qualitative validation of our FASTpp data by comparison to fluorescence data due to several physical differences between the two assays: 1. Heating times (hours in fluorescence, minutes in FASTpp) 2. Fluorescence measures in equilibrium until unfolding and aggregation start while FASTpp constantly removes unfolded protein from the equilibrium – an effect that increases with **t_m_**. The results of FASTpp agree qualitatively with intrinsic fluorescence analysis of Sortase A variants. We conclude that FASTpp is sufficiently sensitive to detect subtle stability differences caused by point mutations.

### FASTpp is applicable to a wide range of protein folds

**Figure 10:**
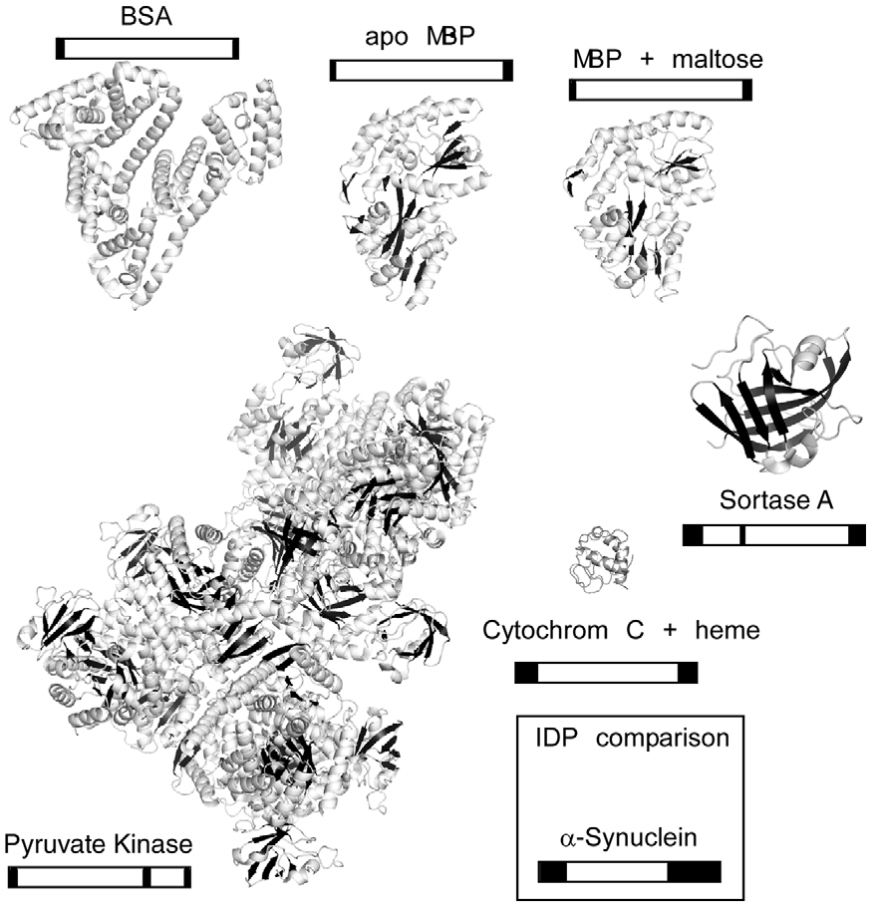
FASTpp is suitable for a wide range of substrates. Representative snapshots from crystallographic studies on the used model proteins. BSA is α-helically folded (pdb identifier 1E7I), MBP has some β-sheets (pdb identifier 1JWY, 1ANF), PK contains more β-sheets (pdb identifier 1F3W), Sortase A mostly β-sheets (pdb identifier 1T2O) and folded Cytochrome C in presence of heme contains extended loops (pdb identifier 1AKK) [27], [28], [34], [46]. The PONDR-FIT predictions are shown in black frames in a simplified view with black indicating a score for intrinsic disorder above 0.5 and background color scores from 0 to 0.5.

To reconcile our data in structural terms, we assessed the structure elements of the proteins analysed by FASTpp and compare these with our metapredictions of structural disorder using the PONDR-Fit algorithm in a simplified dichotomic representation discriminating well-structured/ordered and disordered regions (Fig. 10) [24]. A broad range of folds compatible with the assay: all α-helical, α/β and mostly β-sheet [25]–[28]. BSA is an example for a mostly α-helical protein containing multiple disulfide bonds. Also cytochrome C in the presence of heme as well as MBP contain a large α-helical fraction while cytochrome C in the absence of ligand was previously reported to be largely devoid of structure [14]. Pyruvate kinase forms a 240 kDa complex with somewhat higher β-sheet content [28]. The mostly β-sheet Sortase A protein was amenable to FASTpp analysis as well. This comparison of folds suggests that most folded domains without large internal disordered linkers may be amenable to analysis by FASTpp. Conversely, proteins containing large internal disordered regions are expected to be cleaved by default – unless they fold for instance by a coupled folding and binding mechanism *in vivo* [29]. Accurate disorder predictions for water-soluble proteins such as PONDR-Fit might therefore be useful to preselect suitable candidate proteins for FASTpp assays and guide the data interpretation.

## Discussion

We established FASTpp as a biophysical tool to monitor structural protein stability for both isolated proteins and in lysate. We observed high intrinsic protease activity over a large temperature range from physiological temperatures to 80°C in agreement with previous related studies [11], [30], [31]. An even more thermostable TL variant may extend FASTpp to extremely thermostable substrates [32].

We investigated possible applications of FASTpp for interactions of a folded protein with ligand in either presence or absence of cellular lysate. We obtained an about 10°C higher temperature of unfolding for the ligand saturated MBP in both cases. This agrees qualitatively with previous DSC studies, where MBP unfolded at 55°C and 65°C in maltose-bound form at a heating rate of 1°C/min [16]. It also agrees qualitatively with our data obtained by intrinsic protein fluorescence. The differences of absolute values are likely due to different timescales of heating and the fact that unfolded protein is removed from the equilibrium in the FASTpp assay. Presence of lysate had a stabilising effect on *apo* MBP as monitored by FASTpp while in case of RNAse H stability analysis by Pulse Proteolysis, diluted lysate did not affect the protein stability, possibly due to dilution by urea [1].

Can we determine absolute thermal melting points (T_m_) of proteins by FASTpp? The determination of absolute T_m_ values requires equilibrium conditions, which can be achieved in particular by calorimetric methods [11]. In FASTpp, the unfolding temperature values depend on the experimental conditions such as temperature range, heating rates, protein concentration and protease susceptibility of the protein of interest. While this prohibits determination of absolute T_m_ values, FASTpp accurately determines the relative stability. This allows the precise relative stability analysis of point mutations, ligand binding and different environments including cell lysates [33]–[40].

What method should be chosen for which application? Fluorescence is widely used due to its high sensitivity and in many cases sufficient intrinsic label concentrations of either naturally occuring tryptophanes or genetically engineered fluorescent tags [6], [7], [41], [42]. FASTpp is a useful complementation to fluorescence-based assays in cases where intrinsic labels are below detection levels or genetic manipulation is not possible. The specific advantage of FASTpp, however, is its ability to analyse protein stability at low concentrations and in complex solutions, such as lysates and primary patient samples. Specific antibodies allow stability analysis by FASTpp of cell or tissue-derived samples without the need for tagging or purification. To investigate possible links between biophysical and pathological mechanisms of tumour mutations, patient tissues may be analysed for putative stability changes in disease-related proteins such as kinases and tumour suppressors [6], [43]–[45]. FASTpp experiments can be done in laboratories equipped with standard biochemistry instruments and do not require advanced biophysical equipment.

FASTpp is also an alternative for Pulse Proteolysis. In this *ex vivo* assay, equilibrium unfolding at room temperature in urea precedes a short proteolysis pulse to probe unfolding [1]. Several features of FASTpp differ significantly from Pulse Proteolysis: 1. The rapid temperature increase in FASTpp significantly increases the denaturation rate of kinetically-stable proteins compared to urea titrations at room temperature, e.g. for ligand-bound maltose binding protein [1]. 2. High temperature (up to 80°C) has little effect on the intrinsic proteolysis rate; high urea concentrations however inhibit the enzyme [1]. 3. Temperature gradients reveal quickly self-aggregating unfolded species while urea may dissolve aggregates. Taken together, both approaches have complementary benefits: FASTpp gives insight into thermal stability, Pulse Proteolysis into equilibrium unfolding. FASTpp, however, requires less experimental time.

Considering the broad range of folds that can be analysed by FASTpp and the specificity, robustness and speed of the method, we anticipate a broad range of future applications. Minimal sample preparation requirements and use of standard molecular biological techniques allow applications in protein engineering, cell biology and biomedical research.

## Methods

### Ethics statement

N/A.

### Thermal Proteolysis

We prepared a 5 g/L stock solutions of TL (Sigma) as described earlier [1]. The proteolysis assay buffer contained 10 mM CaCl_2_, 20 mM sodium phosphate buffer at pH 7.2 and 150 mM NaCl for purified proteins and 5 mM DTT for cytosolic proteins. Protein concentrations were between 0.15–1 g/L. Digestion was performed in a C1000 thermal cycler (Biorad) and protein amounts were quantified by coommassie fluorescence in an Odyssey scanner (LiCor); specific fluorescence enhancement of coommassie upon binding to protein was measured and the integrated fluorescence intensity per protein band was compared to the corresponding two-fold dilution series of undigested proteins of known concentration to fit the parameters of a second-order polynom describing the dependence of fluorescence on protein concentration [23].

### Determination of the temperature dependence of the intrinsic proteolysis rate of TL

We determined temperature dependence of TL activity analogous to a previous approach for monitoring urea dependence of TL activity [1]. Briefly, we used 6 nM and 3 nM TL to cleave a fluorigenic model substrate (ABZ-Ala-Gly-Leu-Ala-NBA) to monitor the reaction by fluorescence dequenching of this substrate at various temperatures. For quantification we used a pseudo-first-order kinetic model that assumes a constant concentration of the catalyst (TL) over the course of the experiment and full accessibility of the substrate. As fluorescence increases relative to the extent of dequench, we fitted the intrinsic rate by using the formula:
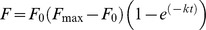
F is fluorescence, F_0_ is the initial fluorescence, F_max_ is the fluorescence after complete cleavage and k is the intrinsic rate of proteolysis at the specific enzyme concentration used, t is the observation time. We fitted the data using Gnuplot.
